# Pre-test metyrapone impairs memory recall in fear conditioning tasks: lack of interaction with β-adrenergic activity

**DOI:** 10.3389/fnbeh.2015.00051

**Published:** 2015-03-03

**Authors:** Mariella B. L. Careaga, Paula A. Tiba, Simone M. Ota, Deborah Suchecki

**Affiliations:** ^1^Departamento de Psicobiologia, Universidade Federal de São PauloSão Paulo, Brazil; ^2^Centro de Matemática, Computação e Cognição, Universidade Federal do ABCSão Paulo, Brazil

**Keywords:** memory, metyrapone, propranolol, fear conditioning, retrieval

## Abstract

Cognitive processes, such as learning and memory, are essential for our adaptation to environmental changes and consequently for survival. Numerous studies indicate that hormones secreted during stressful situations, such as glucocorticoids (GCs), adrenaline and noradrenaline, regulate memory functions, modulating aversive memory consolidation and retrieval, in an interactive and complementary way. Thus, the facilitatory effects of GCs on memory consolidation as well as their suppressive effects on retrieval are substantially explained by this interaction. On the other hand, low levels of GCs are also associated with negative effects on memory consolidation and retrieval and the mechanisms involved are not well understood. The present study sought to investigate the consequences of blocking the rise of GCs on fear memory retrieval in multiple tests, assessing the participation of β-adrenergic signaling on this effect. Metyrapone (GCs synthesis inhibitor; 75 mg/kg), administered 90 min before the first test of contextual or tone fear conditioning (TFC), negatively affected animals’ performances, but this effect did not persist on a subsequent test, when the conditioned response was again expressed. This result suggested that the treatment impaired fear memory retrieval during the first evaluation. The administration immediately after the first test did not affect the animals’ performances in contextual fear conditioning (CFC), suggesting that the drug did not interfere with processes triggered by memory reactivation. Moreover, metyrapone effects were independent of β-adrenergic signaling, since concurrent administration with propranolol (2 mg/kg), a β-adrenergic antagonist, did not modify the effects induced by metyrapone alone. These results demonstrate that pre-test metyrapone administration led to negative effects on fear memory retrieval and this action was independent of a β-adrenergic signaling.

## Introduction

Emotional situations are better remembered than neutral ones because they trigger the release of stress hormones, i.e., glucocorticoids (GCs), adrenaline and noradrenaline, which play a critical modulating role in learning and memory (McGaugh and Roozendaal, 2002; Sandi, 2011). Stressful conditions activate the Hypothalamus-Pituitary-Adrenal (HPA) axis, the main neuroendocrine mediator of the stress response (for review, see Joëls et al., 2012), inducing the release of GCs (cortisol in humans and corticosterone in rodents) by the adrenal glands. GCs act in different brain areas, including the hippocampus and amygdala, which are important for spatial and emotional memory, respectively (Morris et al., 1982; LeDoux, 1992; McGaugh, 2000). GCs effects are mediated by two receptors, the mineralocorticoid (MR) and glucocorticoid (GR) receptors. The former has high affinity for natural GCs and is predominantly occupied under basal conditions, whereas the latter has low affinity for the natural ligand and is occupied at the circadian peak and during stress (Reul and de Kloet, 1985; for review, see Joëls and de Kloet, 1994; Joëls and Baram, 2009). Pharmacological studies using agonists and antagonists for these receptors demonstrate the importance of the MR/GR balance on memory (Oitzl and de Kloet, 1992; Lupien et al., 2002; Rimmele et al., 2010), testifying that both, in some way, are relevant. GCs effects on memory are usually dependent on their levels and the aversiveness of the task. For memory acquisition GCs levels, but not task aversiveness, are essential and an inverted U-shaped dose-response relationship is usually observed (Conrad, 2005). However, for memory consolidation, GCs impact depends on the task aversiveness. Chronic restraint stress before Y-maze (a non-aversive task) impairs spatial memory (Conrad et al., 1996). In turn, infusion of GR agonist into the hippocampus after inhibitory avoidance training (an aversive task) has a facilitatory effect (Roozendaal and McGaugh, 1997). Nevertheless, either high (de Quervain et al., 1998) or low GCs concentrations (Lupien et al., 2002) impair memory retrieval.

During stressful conditions, the adrenergic system is also activated, both centrally and peripherally. Adrenaline is secreted by the adrenal medulla and is the main peripheral product of the sympathetic pathway influencing several central functions such as memory retention, consolidation and information processing (Gold and Van Buskirk, 1975; Dahlgren et al., 1980; Berntson et al., 2003). During or following learning, the increase in GCs or adrenaline concentration by drugs and other treatments enhance memory in rodents and human subjects (Gold and Van Buskirk, 1978; Williams and McGaugh, 1993; Cahill and Alkire, 2003). As adrenaline does not cross the blood-brain barrier, these central effects suggest the existence of a peripheral-central pathway by which adrenaline can modulate processes in the central nervous system (Izquierdo et al., 1959, 1960; Lawrence et al., 1995; Van Bockstaele et al., 1999). After training, administration of noradrenaline or β-adrenergic agonists into the basolateral complex of the amygdala (BLA) improves memory retention on inhibitory avoidance and spatial water maze tasks (Ferry and McGaugh, 1999; Hatfield and McGaugh, 1999) whereas blocking noradrenaline effects with β-adrenergic antagonist propranolol impairs retention (Hatfield and McGaugh, 1999).

Studies reveal that GCs interact with the adrenergic system modulating memory processes specially during stress conditions. Roozendaal et al. studied extensively this interaction and demonstrated that, by acting on the GR, GCs facilitate noradrenergic activation in the nucleus of the solitary tract, which in turn projects to the amygdala and modulates memory consolidation (Roozendaal et al., 1999). Moreover, Quirarte et al. (1997) also showed that the facilitatory effect of the synthetic GC, dexamethasone, on memory consolidation is dependent of β-adrenergic signaling into the BLA, since the administration of β-adrenergic antagonists blocks this dexamethasone effect. The interaction between GCs and the adrenergic system is also extended to memory retrieval. Systemic administration of propranolol (PROP), a β-adrenoceptor antagonist, blocks corticosterone-induced impairment of contextual memory retrieval (Roozendaal et al., 2004a). Likewise, lesion into the BLA or infusion of a β-adrenoceptor antagonist blocks the impairment on memory retrieval caused by GR agonist infused into the hippocampus (Roozendaal et al., 2003). Furthermore, impairment of retrieval in the inhibitory avoidance or fear conditioning induced by restraint stress or GCs administration depends on β_2_-adrenoceptors, which activation decreases cAMP levels, an important intracellular messenger required for the retrieval of hippocampus-dependent memory (Schutsky et al., 2011).

The mechanisms by which high levels of GCs induce deleterious effects on memory were extensively studied in the past decades. Nevertheless, the impairment effect on memory retrieval induced by low levels of GCs is still not well understood. The present study sought to investigate this issue, by using an inhibitor of GCs synthesis (metyrapone; MET) to prevent the hormone elevation in response to re-exposure to the conditioned stimulus. In addition, we also investigated whether the effects induced by MET were dependent on β-adrenergic signaling and if these effects could modify retrieval and subsequent fear memory processing, addressed by a multiple test exposure design.

## Materials and methods

### Subjects

Male Wistar rats, aged 3 months, were obtained from the Center for Development of Experimental Models (CEDEME) of Universidade Federal de São Paulo and used in all experiments. They were kept in groups of 5 rats per cage in a temperature controlled room and maintained on a standard 12:12 light/dark schedule (lights on at 7:00 h) with free access to food and water. Animals were trained and tested during the light phase (between 13:00 h and 16:00 h). All procedures were approved by the University Ethics Research Committee, in accordance with international guidelines for the care and use of animals in research (Protocol N° 2073/11).

### Drugs and administration procedures

Metyrapone (2-metil-1, 2-di-3-piridil-1-propanona; 375 mg in 10 ml; Sigma®), an 11β-hydroxylase inhibitor, was dissolved in vehicle solution containing 40% of propylene glycol and 60% of 0.9% saline to reach the appropriate concentration. The final dose of MET (75 mg/kg) was determined in a previous study (unpublished data).

The β-adrenoceptor blocker Propranolol (Propranolol hydrochloride; Sigma®) was prepared in the same vehicle as MET. Initially, a dose-response curve was performed (10 mg in 10 ml for the 2 mg/kg dose; 25 mg in 10 ml for the 5 mg/kg dose and 50 mg in 10 ml for the 10 mg/kg dose) and the dose of propranolol chosen (2 mg/ kg) was administered on the subsequent experiments.

The drugs and vehicle were administered i.p. in a volume of 2.0 ml/kg. The time of administration of each drug and vehicle is shown on Table 1.

**Table 1:** **Treatment design used in the behavioral experiments**.

Experiment	Administration time	Number of animals/Group						
		VEH	MET	PROP	VEH + VEH	MET + VEH	VEH +PROP	MET + PROP
Effects of acute MET treatment on retrieval and subsequent memory processing of CFC	90 min before Test 1	10	9
	Imediately after Test 1	10	10
Effects of acute PROP treatment on retrieval and subsequent memory processing of CFC	30 min before Test 1	9		PROP2 mg/kg (8) PROP5 mg/kg (8) PROP10 mg/kg (9)
Effects of combined acute MET and PROP on retrieval and subsequent memory processing of CFC	MET (90 min) and PROP (30 min) before Test 1				10	8	9	9
	Imediately after Test 1				8	7	10	9
	Four days after training without Test 1				9	8	9	8
Effects of acute MET and PROP on retrieval and subsequent memory processing of TFC	MET (90 min) and PROP (30 min) before Test 1				10	8	9
Effects of acute MET on non-associative fear expression	90 min before Test	10	10
MET effect on anxiety-like behaviors and locomotor activity	90 min before Test	5	5

*The memory tasks used, the moment of administration of the drugs and the number of animals per group are shown*.

## Behavioral protocols

### Contextual fear conditioning (CFC)

The training phase was performed in one session using a conditioning chamber (30 × 21 × 30 cm) made by black acrylic walls and metallic grids (0.4 diameter) spaced 1.2 cm each and connected to a shock generator and control module (AVS Projetos—São Paulo, Brazil). Animals were placed individually in the apparatus and remained there for 2 min (habituation). After habituation, rats received 5 foot shocks (0.8 mA; 1 s duration and 30 s interval), being removed from the apparatus 1 min after de last one. Freezing behavior, defined as complete immobility of the animal with the absence of vibrissae and sniffing and maintenance only of breathing movements (Fanselow and Bolles, 1979), was measured continuously and was used as measurement of animal’s fear response. Four, 8 and 12 days after training (Tests 1, 2 and 3, respectively), the animals were placed back in the conditioning context and freezing was recorded for 5 min. Data were expressed as percentage of time spent in freezing. The context chamber was cleaned with alcohol 70% diluted in water between animals.

### Tone fear conditioning (TFC)

The training phase was performed in one session using the same chamber of contextual fear conditioning (CFC). Animals were placed individually in the apparatus and remained there for 2 min (habituation). After habituation, rats received 5 tone-shock pairings (tone: 80 dB, 5 s duration; shock: 0.8 mA, 1 s duration and 30 s interval; shock and tone terminated together), being removed from the apparatus 1 min after the last pairing. In testing sessions, a white cylindrical chamber (35 cm diameter, 60 cm height) with an acrylic lid was used. Rats were placed in the apparatus and the tone was presented 5 times in the absence of shocks, but replicating the training schedule. The tests sessions were conducted in a different room from that where training was carried out. Freezing was recorded for 5 min and was used as measurement of animal’s fear response. Data were expressed as percentage of time spent in freezing. The training and test chambers were cleaned with alcohol 70% diluted in water between animals.

### Non-associative fear response

To address a possible effect of MET treatment on fear expression, the animals’ spontaneous fear response was evaluated. The animals were individually placed in a cylindrical chamber (35 cm diameter, 60 cm height), and remained there for 2 min (habituation). Immediately after, rats were exposed to 3 tone presentations (tone: 80 dB, 10 s duration, 50 s inter-tone interval). To increase the aversiveness of the arena four 60-watt bulb lids were turned on during the entire test. Freezing was recorded for 5 min and was used as a measurement of animal’s fear response. Rats were treated with VEH or MET 90 min before the test. Data were expressed as percentage of time spent in freezing. Between animals, the chamber was cleaned with alcohol 70% diluted in water.

### Open field test

MET effect on locomotor activity and anxiety-like behaviors were tested in the Open Field apparatus. The apparatus consisted in a 80 cm diameter cylindrical arena, divided in three concentric circles, each subdivided in quadrants. The animals were individually placed in the center of the arena and allowed to freely explore it for 5 min. The time spent in the center or peripheral areas, besides the total number of squares crossed, were measured as indicators of anxiety-like behavior and locomotor activity, respectively. Rats were treated with VEH or MET 90 min before the test. Between animals, the chamber was cleaned with alcohol 70% diluted in water.

### Blood sampling and corticosterone assay

Thirty min after Test 1, animals were decapitated and their blood was collected in cooled vials containing EDTA. These samples were centrifuged at 2300 rpm for 20 min at 4°C. Plasma was collected and frozen at −20°C. CORT levels were determined in duplicate by a double antibody radioimmunoassay (RIA) method, using a commercial kit (ICN Biomedicals, Costa Mesa, CA). The sensitivity of the assay is 3.125 ng/ml and intra—and interassay variations are, respectively, 10.3 and 7.1%. The method used was a modification from the original developed by Thrivikraman et al. (1997).

### Statistics

Data are presented as mean ± SEM and were analyzed with Statistica 7®. Normality and sphericity were verified. Outliers (values above 2 S.D.) were discarded from the study. Freezing time was analyzed by repeated measures ANOVA, with Treatment as the between factor and Test (Test 1, Test 2, Test 3) as the within factor. Student’s *t* test and One-way ANOVA were also used to analyze the data from Test 1, tone fear conditioning (TFC), Open Field and non-associative tests results.

The relevance of differences was verified by Cohen’s *d* effect size, using the effect size calculator available at the University of Colorado website.^1^

*Post hoc* analysis was performed by Newman-Keuls, for repeated measures analysis, or Duncan, for non-repeated measures analysis. The significant level for statistical differences was set at *P* ≤ 0.05.

## Results

### Effects of acute MET treatment on retrieval and subsequent memory processing of CFC

#### MET administration before Test 1

**Figure 1:**
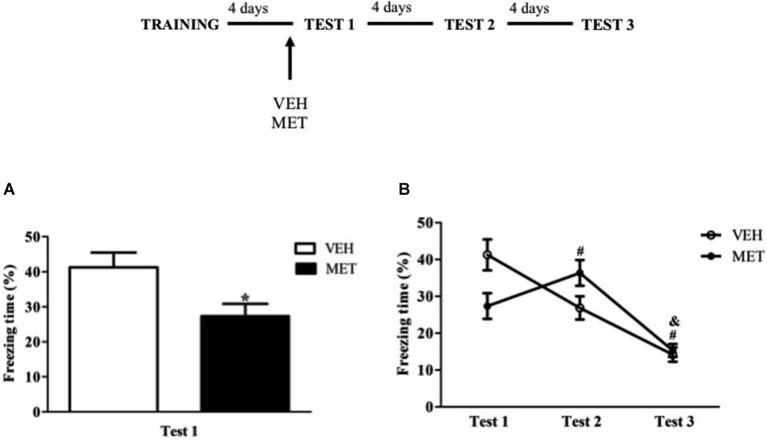
**Acute metyrapone treatment reduced freezing behavior on Test 1.(A)** Metyrapone significantly reduced freezing on Test 1. **(B)** Metyrapone reduced freezing on Test 1 and this effect did not persist on subsequent tests. MET = metyrapone group (*n* = 9); VEH = vehicle group (*n* = 10). *P < 0.05, compared to vehicle group; # *P* < 0.05, compared to Test 1, for all groups; & *P* < 0.05, compared to Test 2, for all groups.

To test the immediate effects of MET administration on contextual memory retrieval, the animals were treated with MET or VEH 90 min before the test. Pre-test MET-treated rats displayed less freezing than VEH group (*t*_17_ = 2.50; *P* < 0.03) (Figure 1A). This difference between the groups also showed a large effect size (MET × VEH, *d* = 1.16).

When all three tests were analyzed, repeated measures ANOVA showed a main effect of Test (*F*_(2,34)_ = 44.43; *P* < 0.01) and a Test × Treatment interaction (*F*_(2,34)_ = 13.91; *P* < 0.01), in which freezing behavior of MET-treated group was higher on Test 2 than on Tests 1 and 3 and VEH-treated group displayed more freezing on Test 1 than on subsequent tests (*P* < 0.01 for both groups and tests) (Figure 1B).

#### MET administration after Test 1

**Figure 2:**
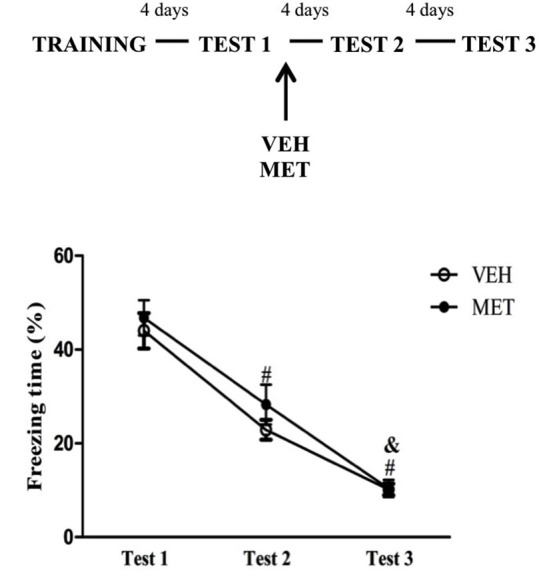
**Post-reactivation acute metyrapone treatment did not modify fear memory expression**. Metyrapone treatment immediately after Test 1 did not modify fear memory expression. MET = metyrapone group (*n* = 10); VEH = vehicle group (*n* = 10). # *P* < 0.05, compared to Test 1, for all groups; & *P* < 0.05, compared to Test 2, for all groups.

To examine whether MET treatment interferes with post-reactivation processes the animals were treated immediately after Test 1. Repeated measures ANOVA revealed a significant effect of Test (*F*_(2,36)_ = 172.18; *P* < 0.01), in which freezing behavior on Test 2 was lower than on Test 1 and was higher than on Test 3 (*P* < 0.01 for all comparisons) (Figure 2). There was no effect of treatment or an interaction.

### Effects of acute propranolol treatment on retrieval and subsequent memory processing of CFC

#### Dose-response curve of propranolol administered before Test 1

**Figure 3:**
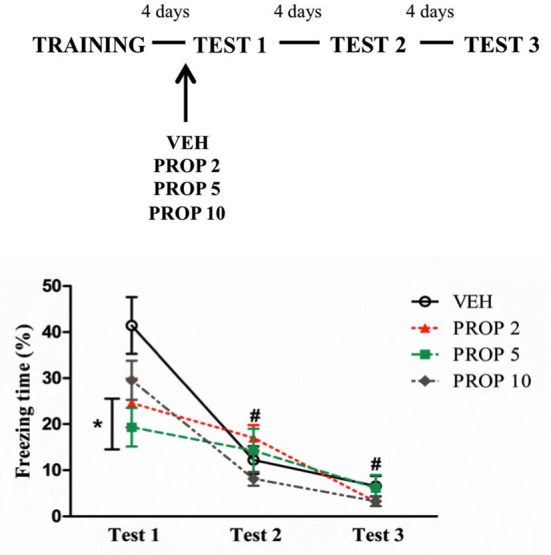
**Acute propranolol treatment reduced freezing behavior on Test 1**. Acute propranolol treatment with 2 or 5 mg/kg before Test 1 reduced freezing behavior. PROP 2 = propranolol 2 mg/kg (*n* = 8); PROP 5 = propranolol 5 mg/kg (*n* = 8); PROP 10 = propranolol 10 mg/kg (*n* = 9); VEH = vehicle group (*n* = 9). *P < 0.05, compared to vehicle group; # *P* < 0.05, compared to Test 1, all groups.

To evaluate the effects of pre-test PROP treatment, the animals were treated with different doses of PROP 30 min before being tested. Repeated measures ANOVA showed a significant effect of Test (*F*_(2,60)_ = 65.68; *P* < 0.01) and an interaction Test × Treatment (*F*_(6,60)_ = 4.25; *P* = 0.01). *Post hoc* analysis showed that PROP 2 and PROP 5 groups displayed less freezing than VEH on Test 1 (*P* = 0.04 and *P* = 0.01, respectively), with large effect sizes (PROP 2 × VEH, *d* = 0.99; PROP 5 × VEH, *d* = 1.42) (Figure 3). VEH and PROP 10 groups displayed more freezing on Test 1 than on Test 2 (*P* < 0.01 for both groups), whereas on Test 3, all groups exhibited a reduction of freezing behavior compared to Test 1 (*P* < 0.05 for all groups) (Figure 3).

### Effects of acute MET and propranolol combination on retrieval and subsequent memory processing of CFC

#### Drugs administration before Test 1

**Figure 4:**
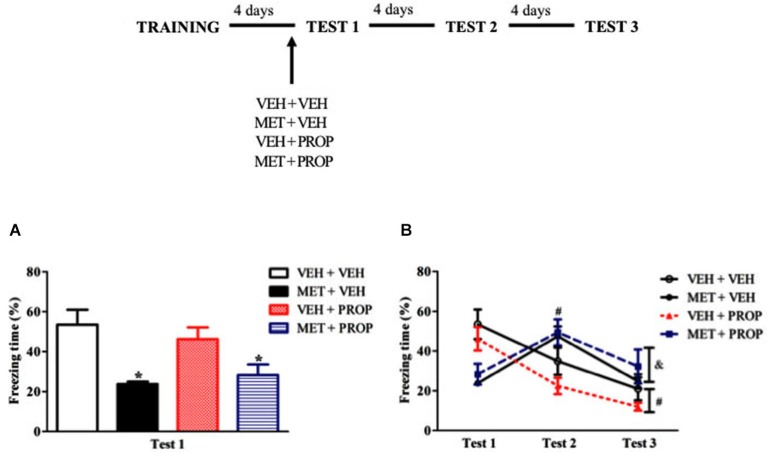
**Acute propranolol treatment did not modify the effect induced by acute metyrapone treatment on contextual fear memory. (A)** Metyrapone treatment significantly reduced freezing on Test 1 and this effect is not modify by PROP treatment. **(B)** Metyrapone treatment 90 min before Test 1 reduced freezing behavior on Test 1 and this effect did not persist on subsequent tests. VEH + VEH = vehicle group (*n* = 10); MET + VEH = metyrapone group (*n* = 8); VEH + PROP = propranolol group (*n* = 9); MET + PROP = metyrapone and propranolol group (*n* = 9). *P < 0.05, compared to control group; # *P* < 0.05, compared to Test 1, for all groups on Test 2 and for VEH + VEH and VEH + PROP on Test 3; & *P* < 0.05, compared to Test 2, for MET-treated groups.

To test whether pre-test MET effects would depend on β-adrenergic signaling, the animals were treated with MET and PROP, respectively, 90 and 30 min before Test 1 in a double i.p. injection design. Based on the dose-response experiment, 2 mg/kg of PROP was chosen. One-way ANOVA for Test 1 showed a significant difference (*F*_(3,34)_ = 6.30; *P* < 0.01) and Duncan test revealed that MET-treated groups exhibited lower freezing than VEH + VEH group (*P* < 0.01) (Figure 4A). Comparison of MET-treated with the other groups also revealed large effect sizes (VEH + VEH × MET + VEH, *d* = 1.76; VEH + VEH × MET + PROP, *d* = 1.26). Interestingly, PROP treatment did not change MET-induced freezing behavior on Test 1 (*P* = 0.72).

Analysis of the three test sessions using repeated measures ANOVA revealed a main effect of Test (*F*_(2,68)_ = 20.83; *P* < 0.01) and a Test × Treatment interaction (*F*_(6,68)_ = 11.84; *P* < 0.01), in which MET-treated rats displayed higher freezing behavior on Test 2 than on Test 1 (*P* < 0.01) and Test 3 (*P* < 0.02), whereas VEH + VEH and VEH + PROP groups displayed less freezing on Tests 2 and 3 than on Test 1 (*P* < 0.05 and 0.01, respectively) (Figure 4B).

#### Corticosterone assay

Analysis of CORT levels 30 min after Test 1 did not detect significant effects (*P* = 0.10). However the comparison between MET and VEH groups revealed a large effect size (VEH + VEH × MET + VEH, *d* = 1.2; VEH + VEH × MET + PROP, *d* = 2.07) (Table 2).

**Table 2:** **Corticosterone plasma levels of animals submitted to different treatments**.

Group	Corticosterone plasma levels (ng/ml)
VEH + VEH	189.92 ± 23.40 (*N* = 4)
MET + VEH	142.28 ± 17.11 (*N* = 3)
VEH + PROP	205.27 ± 63.25 (*N* = 2)
MET + PROP	117.38 ± 7.90 (*N* = 4)

*Blood samples were obtained 30 min after Test 1 and the values are presented as mean ± SEM. The number of animals per group is shown in parenthesis*.

#### Drugs administration after Test 1

**Figure 5:**
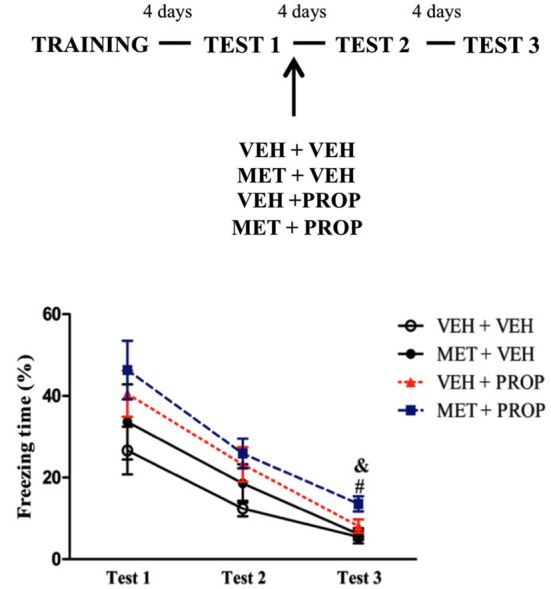
**Acute metyrapone and propranolol treatments immediately after contextual fear memory reactivation did not modify fear memory expression**. There were no differences between the groups when the treatments were administered immediately after Test 1. VEH + VEH = vehicle group (*n* = 8); MET + VEH = metyrapone group (*n* = 7); VEH + PROP = propranolol group (*n* = 10); MET + PROP = metyrapone and propranolol group (*n* = 9). &# *P* < 0.05, compared to Test 1, for all groups; & *P* < 0.05, compared to Test 2, for all groups.

Administration of the drugs immediately after Test 1 revealed that freezing behavior on Test 3 was lower than on Tests 1 and 2 for all groups (*P* < 0.01) (main effect of Test (*F*_(2,60)_ = 63.49; *P* < 0.01) (Figure 5).

#### Drugs administration without re-exposure to the context (Test 1)

**Figure 6:**
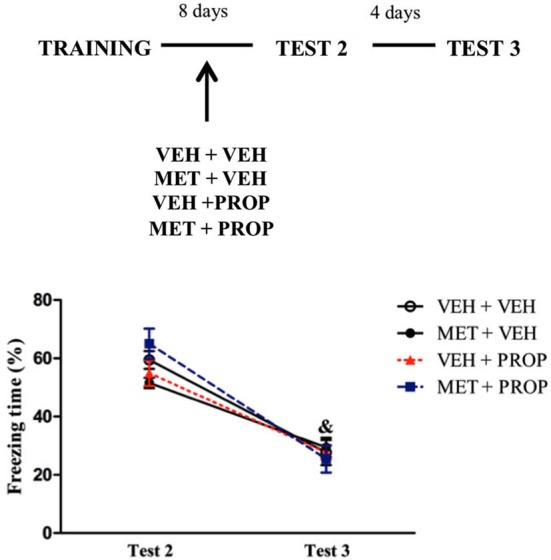
**Acute metyrapone and propranolol treatments without first context exposure did not modify fear memory**. There were no differences between the groups when the treatments were administered without Test 1 re-exposure. VEH + VEH = vehicle group (*n* = 9); MET + VEH = metyrapone group (*n* = 8); VEH + PROP = propranolol group (*n* = 9); MET + PROP = metyrapone and propranolol group (*n* = 8). & *P* < 0.05, compared to Test 2, for all groups.

To determine whether exposure to Test 1 could play a role in the effects observed above, animals were not tested after administration of the drugs and the results showed reduction of freezing time from Test 2 to Test 3 in all groups (*P* < 0.01, for all groups), with main effect of Test (*F*_(1,30)_ = 290.86; *P* < 0.01) and an interaction Test × Treatment (*F*_(3,30)_ = 4.15; *P* < 0.02), in which MET + VEH group exhibited a trend on Test 2 to differ from VEH + PROP group (*P* = 0.07) (Figure 6).

### Effects of acute MET and propranolol on retrieval and subsequent memory processing of TFC

**Figure 7:**
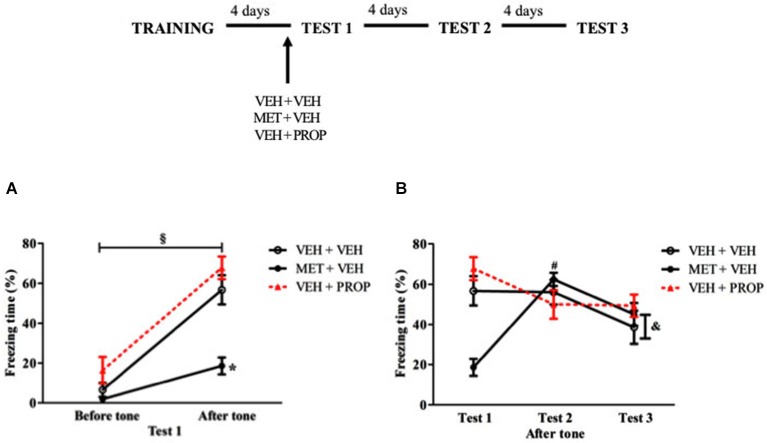
**Acute pre-test 1 metyrapone treatment had similar effects on CFC and TFC. (A)** Metyrapone treatment reduced freezing on test 1 after tone exposure. **(B)** Metyrapone treatment reduced freezing behavior on test 1 but this effect did not persist in subsequent tests VEH + VEH = vehicle group (*n* = 10); MET + VEH = metyrapone group (*n* = 8); VEH + PROP = propranolol group (*n* = 9). *P < 0.05, compared to control group; § *P* < 0.05, comparing pre and post-tone moment. # *P* < 0.05, compared to Test 1, for all groups; & *P* < 0.05, compared to Test 2, only for MET + VEH and VEH + VEH groups.

The hippocampus-independent task was carried out to establish whether pre-test MET or PROP effects would be restricted to contextual memory tasks or not. Analysis of Test 1 indicated that all groups exhibited more freezing after than before tone presentation (*P* < 0.01 for VEH + VEH and VEH + PROP groups and *P* < 0.05 for MET + VEH group), with main effects of Treatment (*F*_(2,24)_ = 12.01; *P* < 0.01), Moment (pre or post-tone) (*F*_(1,24)_ = 135.03; *P* < 0.01) and an interaction (*F*_(2,24)_ = 10.57; *P* < 0.01). Compared to VEH + VEH group, MET + VEH-treated rats displayed less freezing behavior after tone presentation (*P* < 0.01) (Figure 7A). There was also a large effect size when MET + VEH-treated group was compared with VEH + VEH-treated group (*d* = 2.07).

Analysis of the three tests showed main effect of Test (*F*_(2,48)_ = 7.25; *P* < 0.01) and an interaction Test × Treatment (*F*_(4,48)_ = 18.18; *P* < 0.01). MET + VEH-treated group exhibited less freezing behavior on Test 1 than on the other tests (*P* < 0.01 for both comparisons) while PROP + VEH-treated group exhibited more freezing on Test 1 than on subsequent tests (*P* < 0.02 for Test 2 and *P* < 0.03 for Test 3). Finally, VEH + VEH group exhibited less freezing behavior on Test 3 than on tests 1 and 2 (*P* < 0.03 for both comparisons) (Figure 7B).

### Effects of acute MET on non-associative fear expression

**Figure 8:**
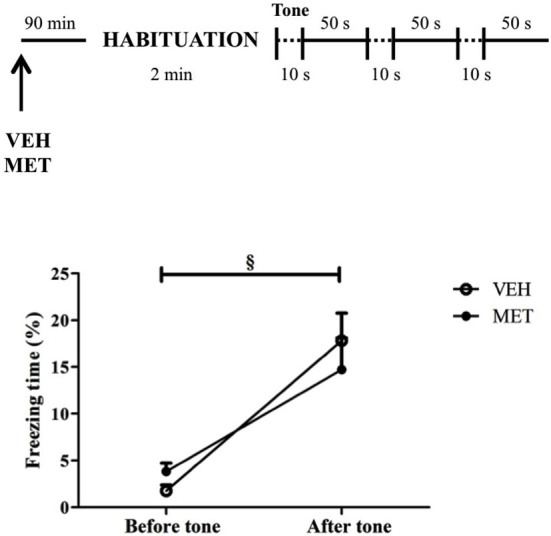
**Acute metyrapone treatment did not impair animal’s ability to express fear**. Metyrapone treatment did not reduce freezing after three unknown and potentially threatening tone presentations. VEH = vehicle group (*n* = 10); MET = metyrapone group (*n* = 10). § *P* < 0.05, comparing pre and post-tone.

This experiment was conducted to investigate the effects of MET treatment on the animals’ innate fear response by assessing the drug effects on a non-associative task. There was a main effect of time, in which animals displayed more freezing after tones (*t*_19_ = 6.82; *P* < 0.001) without differences between the groups on freezing behavior before (*t*_18_ = 1.9; *P* > 0.05) or after tone presentations (*t*_18_ = 0.69; *P* = 0.5) (Figure 8).

### MET effect on anxiety-like behaviors and locomotor activity

**Figure 9:**
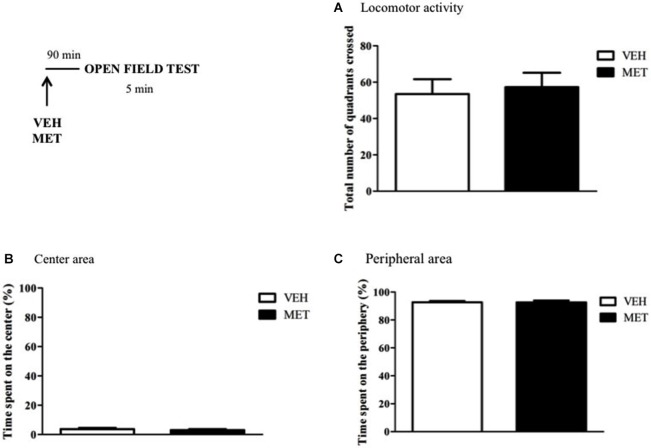
**Metyrapone treatment did not modify animal’s anxiety-like behaviors and locomotor activity. (A)** Metyrapone treatment 90 min before the Open Field test did not interfere with animal’s locomotor activity. **(B)** Time spent in the center of the arena. **(C)** Time spent in the periphery of the arena. VEH = vehicle group (*n* = 5); MET = metyrapone group (*n* = 5).

The effects of MET treatment on anxiety-like behaviors and locomotor activity were evaluated in the Open Field test. MET-treated rats did not differ from VEH-treated ones regarding the total number of quadrants crossed (*t*_13_ = −0.29; *P* = 0.7) (Figure 9A). Furthermore, the time spent in the center or peripheral areas of the apparatus were similar between the groups (Time in the center (*t*_8_ = 0.69; *P* = 0.5); Time in the periphery (*t*_8_ = 0.04; *P* = 0.9)) (Figures 9B,C).

## Discussion

The present study aimed to investigate how blockade of GCs rising in response to an aversive stimulus impacts retrieval and subsequent fear memory processing and whether these effects require adrenergic signaling. MET, a GCs synthesis inhibitor, clearly reduced freezing behavior when administered before the first re-exposure to the conditioned stimulus, suggesting that retrieval of fear memory was impaired in CFC and TFC tasks. The fact that similar results were obtained in both hippocampus-dependent and independent tasks, suggest that MET acts in pathways and/or brain structures common to these tasks and known to present GCs receptors, such as the amygdala (Reul and de Kloet, 1985; Phillips and LeDoux, 1992; Joëls and de Kloet, 1994). The impairment on retrieval induced by MET was restricted to the first test, whereas in the subsequent evaluation (Test 2), in the absence of MET, fear conditioned memory was expressed. In the last test (Test 3), all groups displayed low levels of freezing behavior, indicating that extinction—a process by which the conditioned stimuli no longer predicts the occurrence of the unconditioned stimuli—took place (for review, see Myers and Davis, 2002).

Retrieval impairment in classical conditioning, an example of implicit memory in rodents, and in the radial arm water maze (Salehi et al., 2010), an example of explicit memory, is in accordance with studies in humans showing a dose-dependent effect of GCs on explicit memory tasks (Lupien et al., 2002; Rimmele et al., 2010). Moreover, Marin et al. (2011) also observed that a double, but not a single dose, of MET before retrieval of emotional information impairs this process and this effect persists for 4 days, suggesting a reduction of the strength of the memory trace. Taking together these findings indicate an inverted U-shape curve effect of GCs on retrieval, as proposed for other memory processes. However, the restricted MET effect on Test 1 could be interpreted as a consequence of increased release of corticotropin releasing hormone (CRH), due to reduced GCs negative effect on the HPA axis. This hypothesis is based on evidence showing that CRH impairs hippocampal function and implicit memory (Maras and Baram, 2012). Alternatively, this effect could be due to a nonspecific influence of the drug on freezing expression or on anxiety-like behavior. Regarding the former, MET did not alter the animal’s fear response to an unknown and threatening stimulus compared to VEH-treated rats, indicating that MET impaired memory recall rather than interfered with the animal’s ability to express fear. As for the latter, animals treated with MET 90 min before an Open Field Test session did not display changes in locomotor activity and time spent in the central or peripheral areas of the apparatus, suggesting that MET did not interfere with anxiety-like behaviors, as it is observed with anxiolytic drugs (Treit and Fundytus, 1988).

Behavioral changes induced by MET on Test 2 of both CMC and TFC could also point out to an interference of this treatment on memory, since MET-treated animals displayed more freezing behavior in Test 2 than in Test 1, indicating that memory of the aversive event was preserved and MET-treated groups were unable to access it on Test 1. The hypothesis of retrieval blockade is strengthened by the similar freezing behavior observed on Test 2 in pre-test MET groups and VEH + VEH group, which was not re-exposed to Test 1 (50–55% of freezing time). Moreover, the freezing behavior observed in pre-test MET groups on Test 2 was not expected from animals undergoing extinction process. If that had happened, then MET groups should have displayed freezing behavior similar to VEH group on Test 2. Taking together these comparisons and the non-associative test result, there are strong evidences that pre-test MET treatment had an effect on memory and did not directly affect freezing expression on Test 1. Alternatively, regaining the conditioned response on Test 2 could be interpreted as the result of MET effects on subsequent memory processing, that involves post-reactivation phenomena such as extinction or reconsolidation, triggered by memory retrieval (Nader et al., 2000; for review, see Myers and Davis, 2002). Reconsolidation is a process that maintains or updates the original memory after its destabilization induced by retrieval (Nader et al., 2000), whereas extinction represents the acquisition of new information, an inhibitory learning that reduces the frequency and amplitude of the conditioned response (for review, see Myers and Davis, 2002). Thus, the results of Test 2 could indicate either an impairment of extinction or an enhancement of reconsolidation. Additional evidence of these effects was obtained with MET administration immediately after Test 1 of the CFC, when no differences between groups were observed in subsequent tests, i.e., both groups displayed low levels of freezing, suggesting that memory was not modified and extinction of the conditioned response occurred. The absence of effect induced by MET on post-test 1 condition suggests that the treatment did not interfere with post-reactivation processes, as we previously speculated, reinforcing the idea that MET actions could be restricted to memory retrieval. We should however bear on mind that on post-test 1 experiment perhaps the optimal inhibitory effect of MET, i.e., 90 min after its administration, was not reached, precluding this protocol as an adequate approach for evaluation of a possible modulatory role of GCs on post-reactivation processes. To separate and evaluate each process we should use longer re-exposure duration for extinction analysis and a well-known treatment that interferes with reconsolidation to compare its effects with those of MET. There are studies suggesting a role of GCs or stress on memory extinction and reconsolidation. Barret and Gonzalez-Lima (2004) showed that MET influences extinction of TFC, using an extinction training design and treating animals 90 min before training. Blundell et al. (2011) also demonstrated that corticosterone facilitates extinction while MET prevents it. GR activation by a specific agonist or by dexamethasone prior to extinction training facilitates this process in a dose-dependent manner (Yang et al., 2006). For memory reconsolidation, Maroun and Akirav (2008) showed that exposure to a stressor (elevated platform) immediately after memory reactivation impairs memory reconsolidation, being this effect reversed by GR antagonist infusion into the BLA.

Pre-test administration of PROP also impaired memory retrieval on Test 1 at 2 and 5 mg/kg, corroborating previous studies showing deleterious effects of this drug on spatial memory retrieval in rodents (Murchison et al., 2004). The importance of the adrenergic signaling for retrieval in hippocampus-dependent tasks is also demonstrated in a study with dopamine β-hydroxylase gene knockout mice (Murchison et al., 2004). Administration of β_1_-adrenergic antagonists such as betaxolol also impairs retrieval, suggesting that β_1_-adrenergic receptors are important for this memory process (Schutsky et al., 2011). In the last test (Test 3) all groups treated with PROP showed low freezing behavior, similar to VEH group, indicating that extinction took place. Ouyang and Thomas (2005) showed that PROP treatment before retrieval reduced freezing behavior, but prevented extinction evaluated on a subsequent test. The divergence between the present extinction data and the aforementioned one could rely on the different PROP isomers/doses and/or animal models used in each study. In our study, PROP treatment did not reduce freezing behavior in the first TFC test and this result is in disagreement with Rodriguez-Romaguera et al. (2009) findings that showed reduced fear expression, without impairment of fear extinction. This difference could be explained by the dose of PROP and/or by the different rat strain used in each study. Although Rodriguez-Romaguera et al’s results suggest a modulation of β-adrenergic signaling on fear expression, Murchison et al. (2004) provide another evidence for the lack of adrenergic influence in this task by showing that dopamine β-hydroxylase gene knockout mice exhibited no difference in TFC as they did in CFC, suggesting a different role of adrenergic signaling in each task. Speculatively, the lack of PROP effect in TFC could be due to the administration of vehicle before propranolol treatment, since in previous studies the drug was administered alone or before any other manipulation, avoiding interferences (Murchison et al., 2004; Ouyang and Thomas, 2005).

Finally, we conducted a set of experiments to test whether MET effects on memory were dependent of adrenergic signaling, by treating the rats with a combination of the drugs. Association of the lowest effective PROP dose (even though in this experiment this dose did not produce any effect) with MET did not change the effects observed with MET alone, suggesting that MET-induced retrieval impairment is independent of a β-adrenergic signaling, contrary to what is proposed for high GCs levels (Roozendaal et al., 2004a; Schutsky et al., 2011). This result corroborates the hypothesis that the interaction between GCs and the adrenergic system involves GR activation, as shown by Roozendaal et al. (2004b). Since MET is known to prevent stress-induced GCs elevation and a 90 min interval after MET effectively inhibits corticosteroid synthesis (Roozendaal et al., 1996; Cordero et al., 2002), activation of GR in our study was unlikely to have occurred. Our corticosterone assay did not reveal the expected differences between MET-treated groups and VEH group probably due to the small sample size. Nevertheless, MET-treated animals exhibited large effect sizes when compared to VEH group, suggesting the existence of a relevant biological difference. Moreover, if there were any interaction between the treatments the selected doses should be sufficient to reveal this effect, since a study that investigated the interaction between glutamatergic and cholinergic systems on classical fear conditioning showed that sub-effective doses of the drugs, which alone did not induce any effect, are sufficient to reveal an interaction (Figueredo et al., 2008). On Test 2, all groups treated with MET showed increased freezing behavior, indicating again that these groups did not expressed fear memory during Test 1, but did so, on Test 2. VEH and PROP-treated animals, in turn, displayed a reduction in freezing in the second evaluation compared to the first, suggesting that extinction process began after the first re-exposure to the context for these groups.

Taking these findings together we suggest that MET treatment impaired context and tone fear memory retrieval in a β-adrenergic signaling independent process. The fact that MET administered immediately after the first context re-exposure or 4 days after training without exposure to this test was unable to induce behavioral changes strongly suggests that pre-test administration acts only on memory retrieval and has no effect on subsequent memory processing. This specific effect on retrieval is reinforced by the absence of difference found on the non-associative test and on anxiety-like behaviors. In the post-test administration experiment, VEH + VEH group showed a percentage of freezing not expected for a control group. Even so the result from this experiment was not invalidated once freezing behavior from VEH-treated group in all other experiments was similar to MET and PROP-treated groups in this experiment (approximately 55%), indicating that, indeed, there were no differences between the groups.

In conclusion, our findings suggest that pre-test MET administration impaired fear memory retrieval, in hippocampus-dependent and independent tasks, and this action was independent of a β-adrenergic signaling. In addition, our results suggest that pre-test PROP treatment also negatively impacts CFC but not TFC.

## Conflict of interest statement

The authors declare that the research was conducted in the absence of any commercial or financial relationships that could be construed as a potential conflict of interest.

## Footnotes

^1^http://www.uccs.edu
