# Corrigendum: Pre-test metyrapone impairs memory recall in fear conditioning tasks: lack of interaction with β-adrenergic activity

**DOI:** 10.3389/fnbeh.2015.00123

**Published:** 2015-05-13

**Authors:** Mariella B. L. Careaga, Paula A. Tiba, Simone M. Ota, Deborah Suchecki

**Affiliations:** ^1^Departamento de Psicobiologia, Escola Paulista de Medicina, Neurobiology of Stress and Stress-Related Disorders, Universidade Federal de São PauloSão Paulo, Brazil; ^2^Centro de Matemática, Computação e Cognição, Universidade Federal do ABCSão Paulo, Brazil

**Keywords:** memory, metyrapone, propranolol, fear conditioning, retrieval

Grant information was missing on the original publication. The updated acknowledgment section is provided below.

## Conflict of interest statement

The authors declare that the research was conducted in the absence of any commercial or financial relationships that could be construed as a potential conflict of interest.

